# Conservation of the behavioral and transcriptional response to social experience among *Drosophilids*

**DOI:** 10.1111/gbb.12487

**Published:** 2018-07-09

**Authors:** R. K. Shultzaberger, S. J. Johnson, J. Wagner, K. Ha, T. A. Markow, R. J. Greenspan

**Affiliations:** ^1^ Kavli Institute of Brain and Mind University of California San Diego San Diego California; ^2^ Laboratorio Nacional de Genomica de la Biodiversidad Centro de Investigacion y de Estudios Avanzados‐Irapuato Guanajuato Mexico; ^3^ Department of Cell and Developmental Biology University of California San Diego San Diego California

**Keywords:** behavior, chemosensation, *Drosophilids*, evolution, experience, gene expression, interspecies variation, neurogenomics, socialization, social interactions

## Abstract

While social experience has been shown to significantly alter behaviors in a wide range of species, comparative studies that uniformly measure the impact of a single experience across multiple species have been lacking, limiting our understanding of how plastic traits evolve. To address this, we quantified variations in social feeding behaviors across 10 species of *Drosophilids*, tested the effect of altering rearing context on these behaviors (reared in groups or in isolation) and correlated observed behavioral shifts to accompanying transcriptional changes in the heads of these flies. We observed significant variability in the extent of aggressiveness, the utilization of social cues during food search, and social space preferences across species. The sensitivity of these behaviors to rearing experience also varied: socially naive flies were more aggressive than their socialized conspecifics in some species, and more reserved or identical in others. Despite these differences, the mechanism of socialization appeared to be conserved within the *melanogaster* subgroup as species could cross‐socialize each other, and the transcriptional response to social exposure was significantly conserved. The expression levels of chemosensory‐perception genes often varied between species and rearing conditions, supporting a growing body of evidence that behavioral evolution is driven by the differential regulation of this class of genes. The clear differences in behavioral responses to socialization observed in *Drosophilids* make this an ideal system for continued studies on the genetic basis and evolution of socialization and behavioral plasticity.

## Graphical abstract

The impact of socialization on aggression and social space requirements varies between closely related Drosophild species, while the mechanism of socialization is conserved (species can cross‐socialized each other).

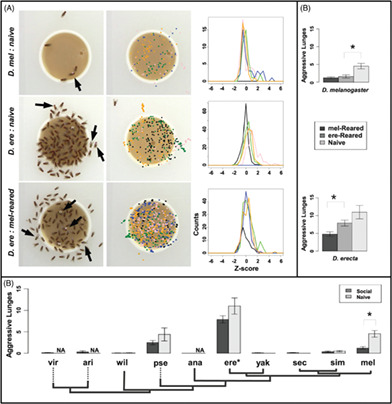

## 1 INTRODUCTION

Prolonged social exposure has been shown to significantly alter the behavior in species ranging from fruit flies to humans,^1^, ^2^, ^3^, ^4^, ^5^ suggesting that the ability to modulate behavior in response to the frequency of social interactions is evolutionarily advantageous. These socially mediated behavioral shifts correlate with experience‐induced alterations in gene expression in the brain,^5^, ^6^, ^7^, ^8^ implying that this behavioral response is partially determined by the properties of the gene regulatory network. One of the clearest examples linking social experience, gene regulation, and behavior is in the fruit fly *Drosophila melanogaster*, where Wang et al. showed that the expression level of a single cytochrome P450 gene, *Cyp6a20*, increases with the amount of time the fly is socialized and is inversely proportional to aggressiveness in males.^5^ Interestingly, *Cyp6a20* is also downregulated in *D. melanogaster* selectively bred to be hyperaggressive,^9^ suggesting that evolution and experience can both modulate behavior through altering the expression levels of the same target genes,^5^, ^6^ and that phenotypic evolution may occur through the canalization of an experience‐altered state.^10^

**Figure 1:**
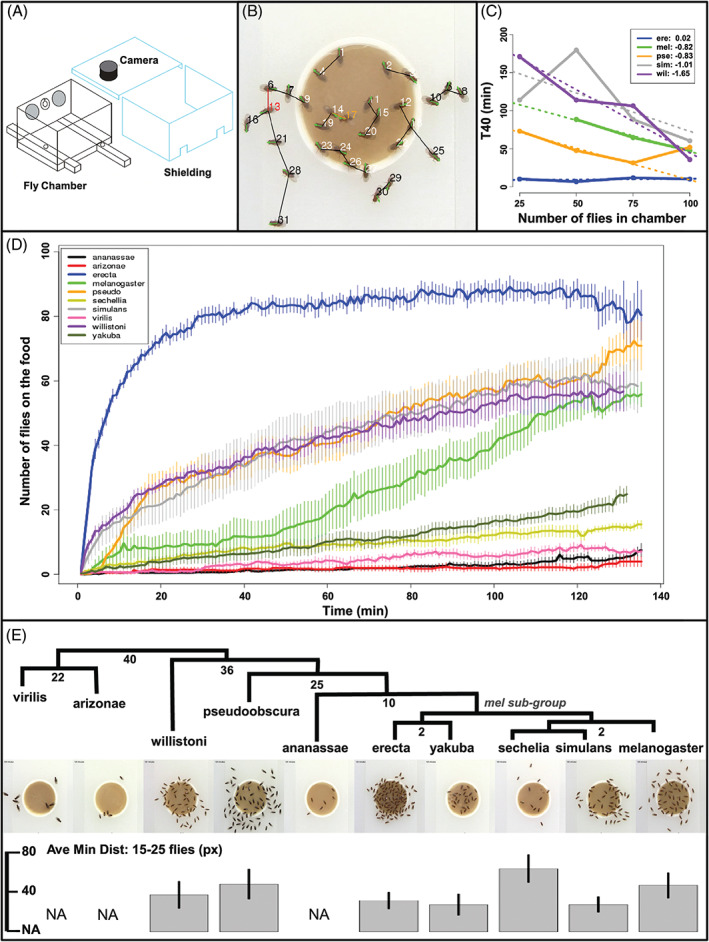
Social feeding behavioral assay. (A) Schematic of social feeding chamber. The shielding was placed around the chamber, and flies were filmed from above. (B) A single, automatically processed frame. Fly number color designates if the fly is on the food (white), off the food (black), or painted (colored). Black line shows distance to nearest neighbor, and green arrows show the predicted orientation of the fly. (C) The amount of time it takes 40% of the population to go to the food source (*y*‐axis) as a function of the number of flies in the chamber (*x*‐axis). Regression lines were fit to the data for each species (dashed lines), and their slopes are reported next to that species in the legend. Each point is the average of at least 4 experimental replicates. (D) Aggregation kinetic plots for each species show the accumulation of 100 virgin females on the food source as a function of time (minutes). Each line is the average of 6 experimental replicates, error bars show ±1 SEM. (E) The phyologenetic relationship between the 10 species used in this study is shown in black. The numbers on the dendrogram are how many millions of years ago those branches diverged based on Matzkin et. al. 20 Below each species is a snapshot of the flies on the food after 120 minutes, and the average minimum distance (social space) ±1 SD calculated from frames that had only 15 to 25 flies (i.e. low‐fly density) during the course of the assay. Social space is measured in pixels (px); *Drosophila melanogaster* average body length = 55.4 px

While many studies have shown that behavior and gene expression are responsive to social experience, the conservation of these effects across multiple species has not been characterized in a controlled assay. Here, we quantified changes in social feeding behaviors across 10 species of *Drosophilids* reared in different contexts (in groups or in isolation), and correlated changes in behavior to gene expression levels in the heads of evolutionarily and experientially diverged flies. We use the term *social feeding behaviors* to encompass behaviors involved in food exploration by a group of flies and interactions among individuals at the food source. Comparisons of independently evolving systems have been extremely useful in the elucidation of biological mechanisms and evolutionary principles.^11^, ^12^, ^13^ This has been especially true in *Drosophilids* where the genomes of more than 30 species have been sequenced allowing for phenotypic differences to be related to genetic changes.^14^, ^15^ For our behavioral comparison, we chose 10 *Drosophilid* species that satisfied the following criteria: (1) have sequenced genomes, (2) cover a large range of evolutionary divergences, (3) are found in contrasting ecologies, and (4) can be reared easily on the same laboratory food source. Eight of these species, *Drosophila melanogaster, Drosophila simulans, Drosophila sechellia, Drosophila yakuba, Drosophila erecta, Drosophila ananassae, Drosophila pseudoobscura,* and *Drosophila willistoni*, belong to the subgenus *Sophophora* and represent both dietary generalists and species that are specialized upon a particular fruit: *D. erecta* on the seasonally restricted *Pandanus* fruit^16^ and *D. sechellia* on the toxic *Morinda* fruit.^17^ The other 2 species, *Drosophila arizonae* and *Drosophila virilis*, belong to the subgenus *Drosophila*, and are separated from the others by approximately 40 million years ago.^14^ Both of these species are specialists: *D. arizonae* breeds in necrotic cactus,^18^ and *D. virilis* breeds in the slime flux exudates of deciduous trees.^19^ The phylogenetic relationships of all 10 species are shown in Figure 1E, and additional ecological information is described in Table S1, Supporting Information.

Social interactions have been shown to be important for food search in *D. melanogaster*. Tinette et al. demonstrated that flies are more likely to go to a food source that already has flies accumulated on it.^21^ This effect was decreased in their assay for vision and smell mutants, suggesting that these social aggregation cues are transmitted through both sensory modalities. Clearly pheromone detection plays an important role in food search as the social pheromone 11‐cis vaccenyl acetate (CVA) has been shown to significantly increase aggregation rates when mixed with food odors.^22^, ^23^ Interestingly, CVA by itself is not sufficient to attract freely searching *D. melanogaster*, but does trigger aggregation in *D. virilis,* 22 suggesting a divergence in how this social cue is processed, and potential interspecies variation in the utilization of social cues during food search.

Once flies aggregate on a food source, they exhibit a range of social interactions including courtship, mating, and aggression.^24^, ^25^, ^26^ These behaviors have been shown to have a wide range of intraspecific variation, but studies that quantify interspecific variation have been limited to a few species, and focused on mating behaviors where large behavioral shifts are thought to drive speciation.^27^, ^28^, ^29^ Interspecific variation in aggression has not been systematically characterized, despite the fact that extensive studies of aggression in *D. melanogaster* have showed that it is a highly variable trait.^9^ These aggression studies have primarily focused on male‐male interactions, but female *D. melanogaster* aggression has been observed and occurs less frequently.^4^, ^30^ Social experience has been shown to alter these interactions in *D. melanogaster*: male courtship and aggression frequencies are reduced after unsuccessful attempts at mating and fighting, respectively,^31^, ^32^ male and female aggression decreases with increased socialization,^4^, ^5^ and social space also decreases with increased socialization.^33^ Conservation of plasticity in these behaviors has not been characterized, but 4 species of *Drosophilids* have been shown to similarly alter their oviposition site preference in the presence of parasitic wasps,^34^ suggesting that specific behavioral responses to experiences can be conserved between species. Furthermore, gene expression levels have also been shown to alter with aggressive and sexual experiences in *D. melanogaster,* 8, 35, 36, 37 but again conservation of experience‐driven gene expression changes and their relationship to behavioral shifts have not been characterized.

## 2 MATERIALS AND METHODS

### 2.1 Food aggregation assay

We adapted the flight chamber described in Tinette et al. for our food aggregation assay^21^ (Figure 1A). The chamber is a Plexiglass box with interior dimensions 12.75″ wide X 8.875″ deep X 10.75″ high. Two flush inset slides in the solid white base of the chamber allow food to be easily introduced and removed in the presence of flies. The box is surrounded on 3 sides by a white plastic shield, and covered by a solid black plastic lid with an access port above each food source that fits a camera lens, or may be closed if no camera is in use. This shielding prevents external visual distractions. A 17″ X 24″ LED lightbox (Artograph A950) was placed 5.75″ from the exposed side of the chamber to provide uniform light during the assay. To further eliminate external distractions, all assays were performed in an enclosed cabinet.

Fly stocks were reared in plastic vials (Genesee Scientific Cat. # 32‐109, El Cajon, California) at 23C on standard lab food made of dark corn syrup (30 mL/L), sucrose (15 g/L), yeast (35 g/L), and agar (10 g/L). A total of 100 virgin females of each species were collected within 20 hours after eclosion and housed in groups of 25 flies. Flies were aged 5 to 6 days, with a target ratio of 50 flies of each age. Flies were maintained on a 12:12 light/dark cycle, and loaded into the chamber at 1 hour after lights on through a load port in the center of the unshielded wall of the chamber (Figure 1A). We acclimated the flies to the chamber for 2 hours prior to food introduction to allow them to recover from the transfer, and to increase their attraction to the food source after a short starvation period. After 2 hours, a white cap (Wheaton Cat. # 239207, Millville, New Jersey) filled with 3 mL of lab food was introduced into the chamber via a slide on the base, and the food source was filmed for at least 2 hours from above with a Canon Vixia HFM301, exposure +1.25, fame rate PF24 (Canon Inc, Melville, New York). Six replicates were performed for each species.

#### 2.1.1 Testing flies reared in different social contexts

To rear socially naive flies, individual pupa cases were transferred to vials separated by cardboard dividers, so emerged flies could not see each other. After these flies emerged, a small dab of acrylic paint was put on the center of their thorax to be able to distinguish them from each other and from socially reared flies in the aggregation assay. These flies were painted red, green, blue, or orange. After painting, they were returned to their individual food vials and maintained in complete isolation. Additionally, a single female was painted white and mixed in with a vial of 25 females as a control to determine if painting affected behavior. A target of 4 socially naive flies, and 1 white control were included as a part of the 100 flies loaded in each assay. To rear individual flies with a different species, single pupa cases were transferred to isolated vials, flies were painted soon after eclosion, and then added to a vial of 25 flies of another species. Immediately prior to assaying, a target of 4 painted flies were aspirated from their respective vials and included in a population of 100 flies with 1 white‐painted control fly. We only tested cross‐rearing between *D. melanogaster* and *D. erecta*. Six replicates were performed for each cross‐rearing experiment.

#### 2.1.2 Food choice and density assays

To assay if food type affects fly aggregation behavior (Figure S1), we measured *D. melanogaster*, *D. arizonae,* and *D. sechellia* on 3 food sources: standard lab food, lab food mixed with cactus rot liquid (natural food of *D. arizonae* 18), or with noni fruit juice (natural food of *D. sechellia* 17). To make the cactus food, we added water to frozen necrotic *Carnegiea gigantea* cactus, incubated for 5 days at 37°C, and added 1 mL of rot liquid to 5 mL of melted lab food. This mixture was then added to a food dish (3 mL) to solidify. To make the noni fruit food, we dissolved 1 g of dried noni fruit leather (Hawaiian Organic Noni, Anahola, Hawaii) in 8.75 mL water at 37°C for 1.5 hours, and added 1 mL of noni juice to 5 mL of melted lab food. This mixture was then added to food dish (3 mL) to solidify. In addition, 3 replicates of the food choice assays were performed with *D. melanogaster*, *D. sechellia*, and *D. arizonae* against all 3 food sources. Fly density experiments were conducted using the standard aggregation assay except that 25, 50, 75, or 100 flies were loaded into the chamber. We performed 4 replicates for each density, and assays ran for at least 2 hours. We were not able to obtain a T‐40 measurement for *D. melanogaster* at a density of 25 flies, because they did not aggregate to a level of 40% over the course of the assay. The regression line for *D. pseudoobscura* was only fit to the first 3 densities. Painted flies were not included for either food choice or dilution assays.

#### 2.1.3 Denatonium assay

To determine how the presence of flies impacts exploration of a food source, we performed a food choice assay similar to the one described by Tinette et al.^21^ We poured two 3 mL food dishes containing lab food. We added 150 μL of 10 mg/mL denatonium to the center of 1 food dish (after solidified), and let sit for 10 minutes. The food aggregation assay was performed as described above, except we slid in 2 food dishes (with and without denatonium) for comparison. The number of new flies was counted that went to each food dish within a 21 minutes window. Four replicates were carried out for *D. melanogaster* and *D. erecta*.

#### 2.1.4 Sensory subtraction assay

To assay the relative importance of vision in socially mediated food search, we poured two 1.5 mL food dishes containing lab food, added 50 virgin females to one, and covered both with a piece of cheesecloth. Both food dishes were loaded into the chamber, and we counted the number of accumulated flies on both dishes after 2 hours. To test the importance of smell, we conducted the same experiment except that both food dishes were covered with a glass coverslip. Three replicates were performed for all conditions for *D. melanogaster* and *D. erecta*.

#### 2.1.5 Cooperative search simulations

Food search simulations were carried out using the script “FlyAggregationSimulation.nlogo” written for Netlogo (https://github.com/shultzab/Fly-aggregation-simulation). Flies searched for a food patch by diffusing through a closed arena, successively turning randomly and moving straight with bouts of random length. Food attractiveness reduces variability of the turns toward the food, and increases as the fly gets closer to the food. When a social interaction parameter is added (cooperativity), food attractiveness increases with the number of accumulated flies on the food. About 100 simulations were performed for all increments of 25 flies between 25 and 200, for both passive diffusion and socially mediated search.

#### 2.1.6 Obp49a mutant assay

To generate a *D. melanogaster Obp49a* heterozygous mutant, we crossed the *Obp49a* deletion strain created by Jeong et al.^38^ (Bloomington Stock #55037, Bloomington, Indiana) with the *D. melanogaster* Canton S strain we used in the species comparisons. As described above, 4 replicates of 100 virgin females aged 5 to 6 days were performed.

### 2.2 Behavioral analysis

Video was automatically analyzed using custom scripts written for R with the EBImage library^39^ (Table S7). All videos were scaled so that the size and position of the food dish were identical for all runs. The number of flies on and proximal to the food dish were counted for every frame over a 10 second window and averaged. This was repeated for every 45 second increment. Social space was calculated as the average minimum distance between all flies within a frame. This was performed automatically by: (1) fitting an ellipse to each fly, (2) finding the nearest neighbor for each ellipse as measured as the distance between ellipse centers, and (3) calculating the mean distance between flies. As social space varied as a function of fly density at the food (more flies trying to eat increased crowding and decreased social space, Figure S6), these means were averaged over all frames that had a specified range of fly densities for interspecies comparison. If 2 flies were the nearest neighbors to each other, that distance was only counted once. To normalize social space to body size for each species, we calculated the mean of the major and minor axes of all fit ellipses in a single frame and subtracted it from the average minimum distance. By doing this correction, social space is a measure of the distance between ellipse edges rather than centers, and is independent of fly size. This independence is confirmed by the lack of correlation between social space measured at a fly density of 15 to 25 flies (Figure 1) and fly body size (*R* ^2^ = 0.19).

To quantify aggressiveness, we manually counted the number of aggressive lunges exhibited by individual flies within their first 150 seconds of arriving to the food source. A lunge is considered aggressive if it displaces another fly, is preceded by display of wing‐threat or reorientation of the fly toward its target, or is accompanied by chasing or multiple rapid contacts.^4^ We scored aggression in all painted flies that went to the food and at least 20 random socially reared flies for each species. Random flies were picked around times that naive flies arrived at the food to compare flies at similar densities. We only scored 9 socially reared *D. arizonae* because they did not aggregate at sufficiently high levels. We were able to score at least 10 socially naive or cross‐species reared flies for each species except for *D. ananassae* (2 naive), *D. arizonae* (1 naive), *D. sechellia* (7 naive), and *D. virilis* (1 naive) because of insufficient aggregation.

#### 2.2.1 Relative positioning of painted flies

**Figure 2:**
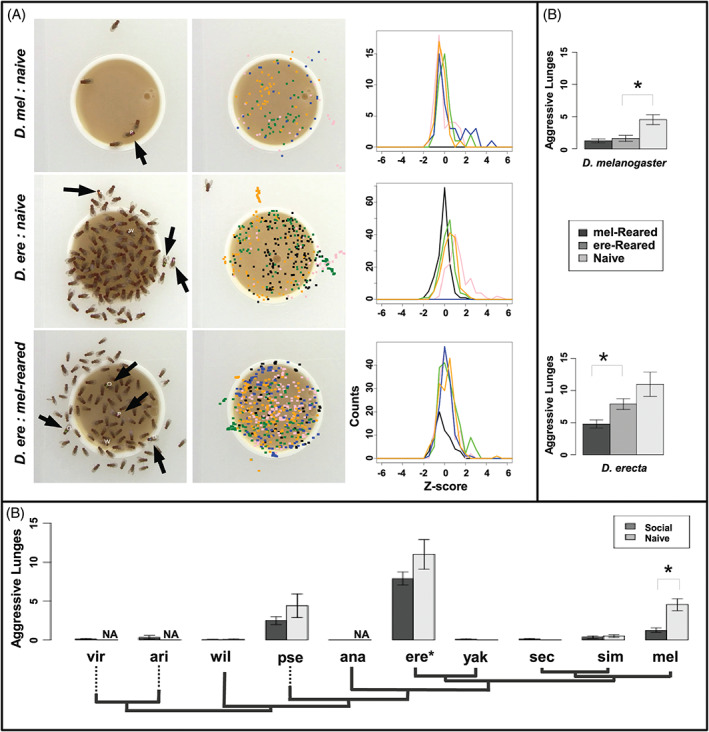
Behavioral responses to rearing context varies across species. (A) Left images: Black arrows show the position of socially naive *Drosophila melanogaster* (top), socially naive *Drosophila erecta* (middle), and *D. melanogaster*‐reared *D. erecta* (bottom). All other flies are socially reared conspecifics. Flies are labeled based on the color of paint on their backs: P‐pink, B‐blue, G‐green, O‐orange, W‐white. White‐painted flies are socially reared control flies. Center images: A single colored square designates the position of each painted fly in a single frame sampled every 40 seconds for the course of the assay. White‐painted control flies are represented as black squares. Right images: Z‐score distributions for each painted fly. A shift in the center of these distribution from 0 to the left or right designates a relatively smaller or larger average social space for that painted fly respectively. These are representative data from a single experiment; 6 replicates were performed for each species. *Z*‐score distributions for all biological replicates are shown in Figure S9. (B) The average number of aggressive events observed in socially reared (social: Dark bars) and isolated (naive: Light bars) flies during the first 150 seconds after they arrive at the food source. The error bars show standard error of the mean. Asterisks designate significant differences. *D. erecta* is significantly more aggressive than all other species. (C) Same as (B) except includes number of aggressive events for flies reared with a different species

By thresholding on color, the position of each painted fly was automatically detected in a single frame and sampled every 40 seconds over the course of the experiment. Each frame was visually inspected, and the positions of painted flies were manually corrected if needed. The distance between a painted fly and its nearest neighbor for each frame was calculated, and the number of SD of that value from the mean of the nearest‐neighbor distribution for all flies in that frame (Z‐score) was determined. A distribution of Z‐scores for all frames for each painted fly was then made to visualize the relative positioning of that fly to the population over the course of the assay (Figure 2A; Figure S9).

### 2.3 RNA‐Seq

#### 2.3.1 Head collection

Virgin females were aged 5 to 6 days in groups of 25 females, and frozen at −80°C in eppendorf tubes for storage. To extract heads, 50 to 100 flies were transferred from −80°C to approximately 50 mL of liquid nitrogen in a 125 mL glass flask. Once the liquid nitrogen evaporated, the flask was rapped forcefully on a pad of paper towels on the bench top approximately 10 times to break the connective tissue holding the fly head to body. Decapitated flies were added to a prechilled metal 0.0278″ sieve on top of a 0.0165″ sieve. This allowed heads to pass through the first sieve, but not the second. Bodies remained on the top sieve, and legs and other random fly parts could pass all the way through the second sieve. For the larger species (*D. virilis*, *D. arizonae*, and *D. pseudoobscura*), we used a 0.0469″ sieve on top which would allow the larger heads to pass through. Heads were then collected in the second sieve, and transferred to a chilled 1.5 mL eppendorf tube for RNA extraction.

#### 2.3.2 RNA‐Seq library preparation and sequencing

To homogenize fly heads, 200 μL of TRI‐Reagent (Ambion, Foster City, California) was added to 50 to 100 heads, and then homogenized with a motorized pestal for 60 to 90 seconds. An additional 800 μL of TRI‐Reagent was added to this homogenate, centrifuged for 1 minute at 12 000*g*, and the supernatant was divided between 2 RNAse free 1.8 mL tubes. Total RNA was then purified using the Direct‐zol RNA MiniPrep kit (Zymo Research, Irvine, California). Total RNA was DNAse treated using the Turbo DNA‐free kit (Thermo Fisher, Waltham, Massachusetts). mRNA purification, mRNA fragmentation, and cDNA synthesis were carried out according to Lott et al.^40^ Illumina RNA‐Seq libraries were prepped using the Illumina TruSeq protocol with barcoded adapters for multiplexing (Illumina Inc., San Diego, California). We multiplexed up to 8 samples for sequencing on a HiSeq 2000 as 50 bp single read runs.

#### 2.3.3 RNA‐Seq processing

Reads were mapped to their respective genomes using CLC Genomics Workbench Version 5.1.5. Up to 2 mismatches were allowed per read. For each species, we used the all_chromosome.fasta files reported on FlyBase^41^ as the reference genome, and the all_filtered.gff files for annotation. The version numbers used for each species are as follows: *D. ananassae* version 1.3, *D. erecta* version 1.3, *D. melanogaster* version 6.02, *D. pseudoobscura* version 3.2, *D. sechellia* version 1.3, *D. simulans* version 1.4, *D. virilis* version 1.2, *D. willistoni* version 1.3, and *D. yakuba* version 1.3. The *D. arizonae* genome was not published, so for this species, we used the closely related *D. mojavensis* version 1.3. Reads per gene were calculated as both raw reads (for use with DESeq^42^) and as RPKMs. RPKM values were subsequently converted to transcripts per million (TPM bases sequenced). To do this, we normalized the RPKM values for all genes, so that their summed value was 1 million.

#### 2.3.4 RNA‐Seq analysis

To identify orthologous genes between species, we used the gene_orthologs_fb_2014_05.tsv gene ortholog assignment reported on FlyBase. For subsequent comparative analysis, we only considered those genes that had an ortholog to a gene in *D. melanogaster*. Table S4 contains the number of genes for each species that had *D. melanogaster* orthologs, the number of reads that mapped to exons in these genes, and the correlation coefficients (*R* ^2^) between all biological replicates. We generated RNA‐Seq data for at least 3 biological replicates for 10 of the 14 conditions assayed (10 species socially reared, 4 species non‐socially reared), and only 2 replicates for the remaining 4 conditions (socialized *D. ananassae*, socialized *D. sechellia*, socialized *D. yakuba* and non‐socialized *D. erecta*). Table S5 has raw read counts, DESeq‐normalized raw read counts (estimateSizeFactors function), RPKM and TPM values for all *D. melanogaster* orthologs in all sequenced samples. To cluster the species based on relatedness in their global gene expression levels, we used the R function **cor** to determine the Spearman rank correlation coefficient for all pairwise species comparisons. We subtracted these values from 1 and used the **as.dist** function to generate a dissimilarity matrix which was then clustered using the **hclust** function with the “Ward” method option.^43^

Differential expression analysis was carried out using the raw read values reported in Table S5 and the DESeq version 1.4.1 functions **newCountDataSet, estimateSizeFactors, sizeFactors, estimateVarianceFunctions** and **nbinomTest** with default parameters.^42^ Differential expression was considered significant if the DESeq reported adjusted *P* value (*padj*) was ≤0.05. Expression in 1 species was considered significantly differentially expressed relative to a set of other species (i.e. the *melanogaster* subgroup) if all pairwise comparisons had a *padj* ≤ 0.05. If 1 species of the set did not have an ortholog assignment for a given gene, that gene was not considered. Significant enrichment of gene ontology terms was carried out using the Princeton Generic Gene Ontology Term Finder found at go.princeton.edu.^44^ To determine if the overlap of differentially expressed genes between samples was significant, we randomly selected the same number of differentially expressed genes for each sample from all *D. melanogaster* orthologs, and calculated the fraction of overlap. We repeated this 10,000 times and determined significance of a given overlap based on the distribution of overlap from the randomly sampled lists.

## 3 RESULTS AND DISCUSSION

### 3.1 Comparative behavioral assay

We quantified changes in social feeding behaviors between evolutionarily and experientially diverged flies using a novel, highly automatable assay (see Section 2). Briefly, 100 virgin females were loaded into a Plexiglass chamber, given a single food source via a slide on the chamber floor, and filmed interacting on the food for at least 2 hours (schematic in Figure 1A). We only used virgin females to limit potential behavioral variability from comparing mated to virgin females (sexual maturation times vary between species^45^). Resulting video recordings were processed to determine: (1) the aggregation kinetics of the flies to the food source, (2) the average distance between each fly and their nearest neighbor (social space^33^, ^46^), (3) the propensity of the flies to be on or near the food, and (4) aggressiveness^4^ (Figure 1B). Aggressiveness was manually scored by counting the number of aggressive lunges exhibited by individual flies within the first 150 seconds of arriving at the food, all other behaviors were quantified automatically using custom image processing scripts written for R (see Section 2). To determine the effects of rearing context on these behaviors, virgins were either collected immediately after eclosion and housed in groups of 25 flies (socialized), or single flies were transferred to individual vials as pupae and raised in complete isolation (non‐socialized). Acrylic paint was placed on the center of the dorsal thorax of isolated females to enable individual tracking among their socialized conspecifics (flies 13 and 17 in Figure 1B are painted). Flies were raised in complete isolation, as opposed to a more “natural” rearing context (small group size or limited social exposure), to minimize the handling of flies prior to behavioral analysis, and to maximize the induced behavioral and transcriptional differences between rearing conditions.

### 3.2 Interspecific variations in group‐reared behaviors

Interspecific differences in social feeding behaviors among group‐reared flies were immediately apparent by examining the density and positioning of each species on the food after 2 hours (Figure 1E). As we only tested a single strain for each species, observed behavioral traits for that strain may not be representive of the species as a whole. Regardless, we provide ecological information for each species to support observed interspecies differences, and to identify behavioral trends across species that evolved under similar environmental pressures. Aggregation kinetics were highly variable, even among closely related species (Figure 1D). *D. erecta*, a dietary specialist whose primary food source (*Pandanus* fruit) is only available 3 months of the year,^16^ aggregated much faster than any other species. This is consistent with previous observations that *D. erecta* aggregate at high densities in the wild,^16^ which suggests that competition for the seasonally restricted *Pandanus* fruit may select for increased aggregation rates in this species. The generalists *D. pseudoobscura, D. melanogaster*, *D. simulans* and *D. willistoni*, which are often found competing with other species for food in nature, also aggregated at high levels in our assay as well as in the wild,^47^, ^48^, ^49^, ^50^ further suggesting that selection in food competitive environments may drive aggregation levels. The remaining species, including the noncompeting dietary specialists *D. arizonae* and *D. sechellia*, aggregated poorly; unlike *D. erecta*, the food source for *D. arizonae* and *D. sechellia* are temporally and spatially abundant. To determine whether food preferences can account for observed differences in aggregation, we tested *D. melanogaster*, *D. arizonae*, and *D. sechellia* on their preferred food source and the preferred food of the other 2 species: standard lab food, cactus rot, and noni fruit, respectively.^17^, ^18^ Species did not exhibit a significant increase in accumulation on their native food (*P* > 0.1; all statistics were performed using the Wilcoxon rank sum test unless otherwise noted), suggesting that variations in aggregation levels are not due to differences in food affinity (Figure S1).

Differences in aggregation may be explained by variability in physiological properties including sensitivity to odors, visual acuity, dehydration rates, and metabolism. Alternatively, these differences may be caused by variations in the use of social cues during food search between species.^21^, ^22^ As previously mentioned, *D. melanogaster* are more likely to go to a food source that already has flies accumulated on it, suggesting that food search kinetics show positive cooperativity.^21^, ^51^ To quantify the degree of social interaction during food search, we measured the amount of time necessary for 40% of the flies in the chamber to go to the food (T‐40) as a function of fly density^51^ (Figure 1C). Of the 5 species that aggregated at high levels in Figure 1D, 4 exhibited a linear decrease in T‐40 as the number of assayed flies increased. This inverse relationship between aggregation time and fly density is predicted by a cooperative search model (Figure S2), and has previously been observed for *D. melanogaster*.^51^ Here, we use the term *cooperative* as it is used in binding kinetics to describe the dependency of food search rate on fly density. We are not supposing that individual flies are actively cooperating to find food. Interestingly, *D. erecta* exhibited no change in T‐40 as a function of fly density suggesting noncooperativity (Figure S2). The degree of cooperativity for an individual species (cooperativity coefficient) can be quantified by taking the slope of the regressions in Figure 1C. *Drosophila melanogaster*, *D. simulans*, and *D. pseudoobscura* show a similar but lesser degree of cooperativity than *D. willistoni*. We only used 3 densities to calculate the correlation coefficient for *D. melanogaster*, which did not aggregate at sufficiently high levels to calculate a T‐40 for 25 flies, and for *D. pseudoobscura*, which did not show a decrease in T‐40 at the highest fly density. The cooperative food search simulation suggests that there is a limit to which an increase in fly number increases the attractiveness of the food (Figure S2). This limit may be lower for *D. pseudoobscura*, which is larger than the other species assayed.

To further assay cooperativity, we performed a food choice assay similar to the one perdomed by Tinette et al.^21^ for *D. melanogaster* (cooperative) and *D. erecta* (non‐cooperative). We presented flies with 2 food sources: one containing the odorless, but taste‐aversive chemical denatonium that prevents aggregation at the food, and one without. As previously observed, the initial kinetics of *D. melanogaster* to either food source was equivalent, but the relative rate of aggregation to the non‐denatonium food increased as more flies accumulated there suggesting cooperative food search (Figure S3).^21^ Like *D. melanogaster*, *D. erecta* showed similar initial kinetics to both food sources, but remained clustered in a small group next to the food containing denatonium, suggesting that even though *D. erecta* may not exhibit cooperativity during food search, they may still be highly social (Figure S4). To determine the importance of vision and smell in our assay, we placed either flies and food, or food alone under either a piece of cheesecloth (no visual cue) or a glass coverslip (no odor cue). No condition was sufficient to drive the aggregation of *D. melanogaster* suggesting a search dependence on both smell and vision (Figure S5), as previously observed by Tinette et al.^21^ *D. erecta* aggregated on either food, or food and flies under cheesecloth at similar rates, suggesting that food odors are the primary attractant for these flies, which is consistent with their apparent loss of cooperativity during food search. *D. erecta* did not accumulate on flies and food covered by a coverslip (Figure S5).

Once aggregated, the species varied significantly in how they interacted on the food. *D. melanogaster*, *D. pseudoobscura*, and *D. sechellia* had a significantly larger social space than all other species at low fly densities (*P* < .01 for all pairwise comparisons), and *D. erecta, D. yakuba*, and *D. simulans* consistently had a smaller social space across densities (Figure 1E; Figure S6). *D. erecta* was significantly more aggressive than any other species (*P* ≤ .05, Figure 2B; Video S1), including its close relative *D. yakuba* which exhibited slow aggregation kinetics and minimal aggressive contact (Figure 1D and 2B). Both species exhibited a strong preference to be on the food (Figure S7) and a tight social space suggesting that aggressiveness and social space can evolve independently. While aggression levels have been shown to be highly variable in *D. melanogaster* males,^9^ the hyperaggressive behavior observed in *D. erecta* has not been observed previously in females. *D. pseudoobscura* was significantly more aggressive than the remaining species except for *D. melanogaster* (*P* ≤ 0.05), which in turn was significantly more aggressive than all other species except for its close relative, *D. simulans* (*P* ≤ 0.05, Figure 2B). As species that aggregated at higher levels in Figure 1D tended to be more aggressive, we tested whether the proportional instance of aggression increased with fly density. It did not for any species (Figure S8), suggesting that like higher aggregation levels, increased aggression may be selected for in food‐competitive species.

### 3.3 Interspecific differences in behavioral response to socialization

Social space and aggression have both been shown to vary with rearing context in *D. melanogaster,* 4, 5, 33 but conservation of these experience‐modulated behavioral shifts had not been characterized. We measured the impact of socialization on these 2 behaviors in all 10 species. As previously observed, *D. melanogaster* flies were significantly more aggressive when raised in isolation (*p* = 5.7*e* ^−5^, Figure 2A,B, Video S2).^4^, ^5^ This trend was consistent but not significant for *D. erecta* and *D. pseudoobscura* (Figure 2B). Socialization did not affect aggression levels in the other species, clearly showing interspecific variation in the plasticity of this behavior. Socially naive *D. melanogaster* did not exhibit a larger social space as previously observed in a pyramid assay,^33^ but *D. erecta* did; naive *D. erecta* went to the food but tended to stay on the outside of the group (Figure 2A, Figure S9, Video S1). This spatial separation was not observed in any other species. Differences in the behavioral response to rearing context could be the result of interspecific differences in the transmission of socialization cues (pheromone profiles vary across species^52^), or interspecific differences in how the received cues feed into downstream behavioral processes. To try to separate these 2 possibilities, we reared individual *D. melanogaster* and *D. erecta* flies in vials with 25 flies of the other species, and tested them in our assay. Cross‐species reared *D. melanogaster* and *D. erecta* were both significantly less aggressive than conspecifics raised in isolation (*P* ≤ 0.005, Figure 2C), indicating that the mechanism of socialization is conserved between these species. *D. erecta* reared with *D. melanogaster* no longer stayed on the outside of the group as seen in socially naive flies further supporting cross‐species socialization (Figure 2A). Interestingly, *D. erecta* raised with the less aggressive *D. melanogaster* were themselves significantly less aggressive than *D. erecta* raised with conspecifics (*P* = 0.03), suggesting socialization cues may be expressed at higher levels in the larger *D. melanogaster*.

### 3.4 Interspecific differences in brain transcriptome

**Figure 3:**
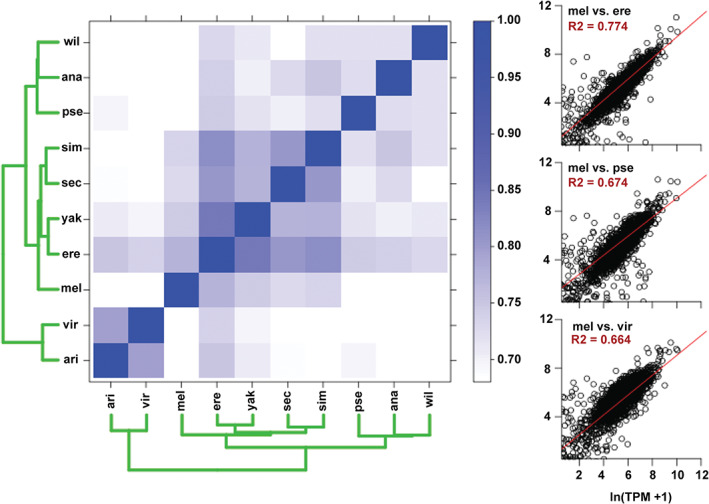
Gene expression levels are moderately conserved across species. Scatter plots on the right show the mean expression level (TPM) for each gene in *Drosophila melanogaster* (*x*‐axis) vs the mean expression level for the corresponding ortholog in *Drosophila erecta*, *Drosophila pseudoobscura*, or *Drosophila virilis* (*y*‐axis). A regression line was fit to these plots for all pairwise species comparisons, and the *R* ^2^ was used to color the heat map on the left (color scale to the right of the heat map, all values below *R* ^2^ = 0.68 are colored white). The green dendrograms show the predicted relatedness of the species to each other as determined by hierarchical cluster analysis (see Section 2)

**Figure 4:**
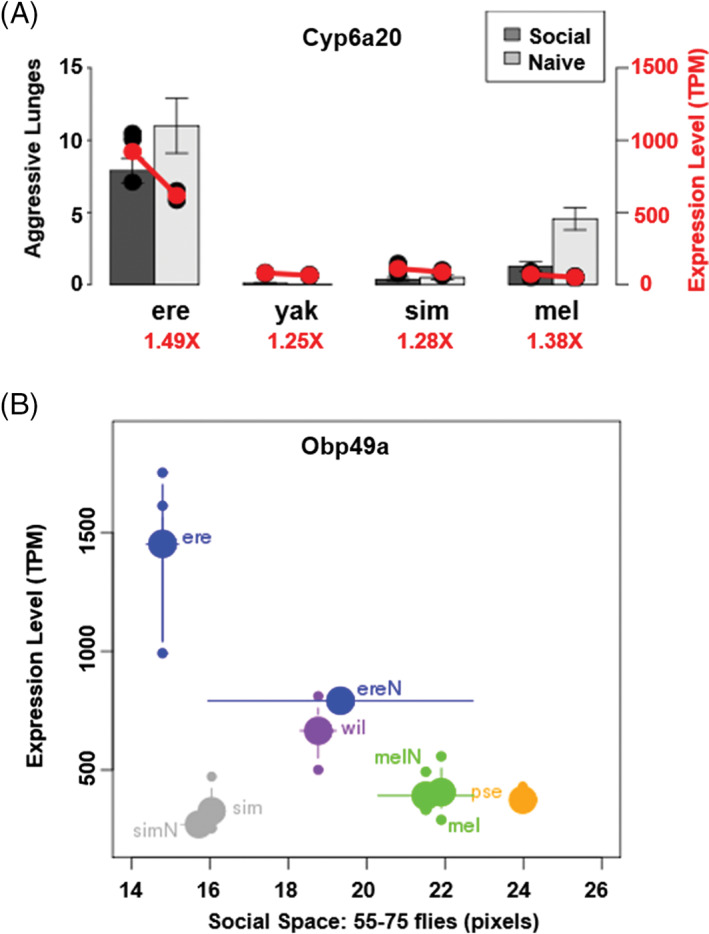
Behavioral variation correlates with gene expression changes. (A) Red circles mark the mean *Cyp6a20* expression level (TPM reads) of biological replicates (black circles) for that species and rearing context. Aggression and *Cyp6a20* expression levels are significantly higher in *Drosophila erecta* relative to all other species. The red numbers under each species name signifies the‐fold change in *Cyp6a20* expression between rearing conditions. (B) Larger circles signify the mean *Obp49a* expression level and smaller circles signify values from each biological replicate. Circles are colored based on the species name next to the large circle. Names that end in “N” signify data for flies reared in isolation (socially naive). Horizontal error bars are the SE of the mean for social space measurement (pixels)

To identify potential genetic effectors of these behaviors and conserved transcriptome changes related to socialization, we measured gene expression levels in the heads of all 10 species reared in groups, and in socially naive *D. melanogaster*, *D. erecta*, *D. simulans* and *D. yakuba*. We focused on experience‐driven transcriptome changes in these 4 species in the *melanogaster* subgroup because they significantly varied in their behavioral response to socialization, and diverged relatively recently (Figure 1E). Gene expression levels were fairly well conserved between species (Figure 3), and clustered roughly as expected based on the previously reported genomic‐sequence derived phylogeny^20^ (Figure 1E). As *D. erecta* exhibited extremes of all behaviors assayed (fast aggregation kinetics, hyperaggression, noncooperative food searching and tight social space), we determined which genes were differentially regulated in this species relative to the rest of the *melanogaster* subgroup (Table S2). Again, we limited our analysis to these species because they significantly varied in behavior and diverged relatively recently. Cytochrome P450 genes, which are involved in pheromone synthesis and degradation,^53^ were significantly enriched among differentially express transcripts in *D. erecta* (*p* = 4.09*e* ^−5^, GO analysis^44^). This included *Cyp6a20*, which is thought to be involved in the degradation of aggression‐triggering pheromones,^9^ and whose expression is inversely related to aggression levels in *D. melanogaster* males as previously mentioned.^5^ Interestingly, *Cyp6a20* was significantly upregulated in the hyperaggressive *D. erecta*, which is counter to the expression‐behavior trend observed in *D. melanogaster*. An evolved upregulation in expression may be advantageous to deal with increased pheromone exposure in the densely packed *D. erecta*. As in *D. melanogaster* males,^5^ *Cyp6a20* levels did increase with socialization in the 4 species where we have expression data for both rearing contexts, but these expression differences were not significant (Figure 4A). However, we did observe a greater relative change in *Cyp6a20* expression in the species that exhibited behavioral sensitivity to rearing condition (*D. erecta* and *D. melanogaster*, Figure 4A). Interestingly, the autism‐linked *Wac* gene, which has been shown to affect nonassociative conditioning in *D. melanogaster*, was also significantly upregulated in *D. erecta* relative to the rest of the *melanogaster* subgroup (Table S2).^54^

Genes involved in chemoreception and processing in the peripheral nervous system (PNS), like *Cyp6a20*, are generally thought to be under strong selection during behavioral evolution.^55^, ^56^, ^57^ The PNS‐expressed odorant‐binding protein (*Obp*) genes, which specifically shuttle odorants and pheromones to odorant receptor neurons,^58^ were also enriched among differentially expressed transcripts in *D. erecta* (*P* = 0.007). This is consistent with the observation that this class of genes is evolving at a faster rate in *D. erecta* relative to the rest of *melanogaster* subgroup, likely due to selection for a specialized diet.^56^ As food search and social interactions are both largely mediated by the perception of chemical stimuli,^55^ it is unclear if behavioral differences in *D. erecta* were specifically selected, or are merely a byproduct of genetic changes during diet specialization. Interestingly, *D. sechellia*, the other dietary specialist in the *melanogaster* subgroup, exhibited social behaviors on the opposite extreme of the phenotypic spectrum (slow aggregation kinetics, nonaggressive, and large social space, Figure 1), suggesting changes in social interactions may indeed accompany large shifts in food preference. Cytochrome P450 genes were enriched among differentially expressed transcripts in *D. sechellia* relative to the rest of the *melanogaster* subgroup (*p* = 5.81*e* ^−8^), but *Obp* genes were not (only 6 *Obp* genes were differentially expressed). However, significant enrichment of *Obp* genes among differentially expressed transcripts in *D. sechellia* relative to members of the *melanogaster* subgroup has been observed when expression differences were limited to the antennae.^59^

### 3.5 Conservation of experience‐driven changes in gene expression

**Figure 5:**
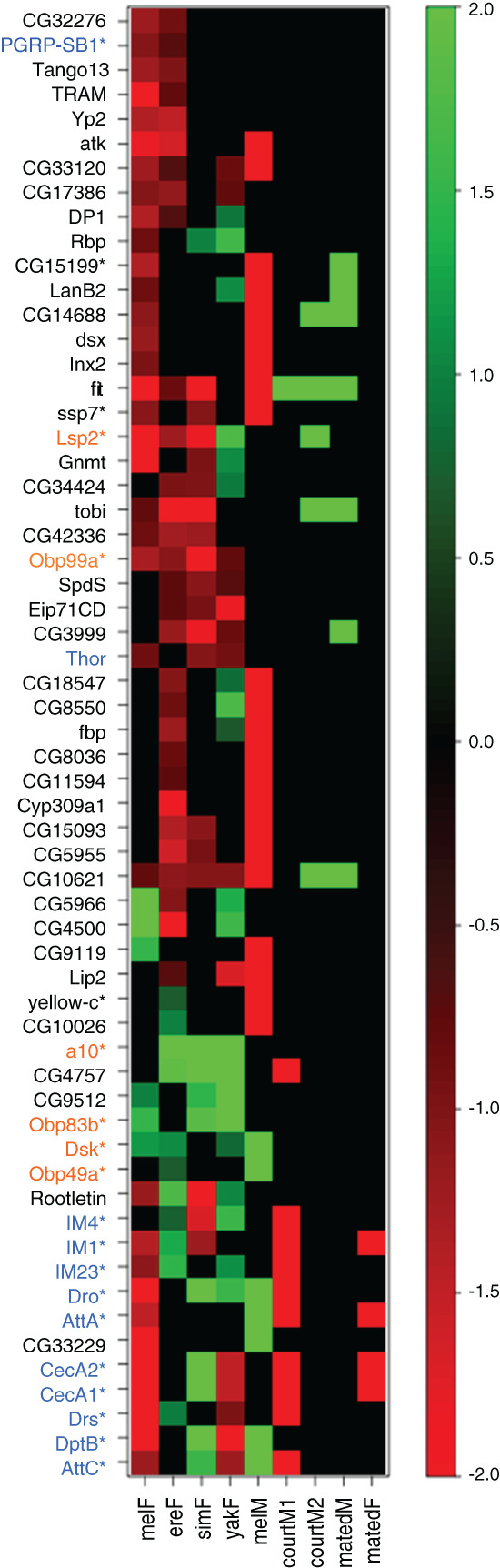
Conservation of experience‐driven expression changes. “melF”, “ereF”, “simF”, “yakF” are the log2 expression change of that gene in socially reared virgin females/socially naive virgin females (color scale on right, values outside of range are set to −2 or 2 depending on sign). If the expression change for a gene in a given condition is not significant, it is black. The remaining columns indicate if that gene was previously identified as significantly altered in *Drosophila melanogaster* after some social experiences. Green is an upregulation and red is a down‐regulation of the first social condition relative to the second in all cases. melM: males raised in groups vs males raised in isolation,^5^ courtM1: males after courting females vs non‐courting males,^35^ courtM2: males after courting females vs non‐courting males,^36^ matedM: mated males vs non courting males,^36^ matedF: mated females vs virgin females.^60^ We show those genes that satisfy at least 1 of the following criteria: significantly altered in: (1) both melF and ereF, (2) melM and either melF or ereF and (3) at least 3 of melF, ereF, simF, and yakF. Orange names are involved in sensory perception, behavior, neuro‐signaling, and neurodevelopment. Blue names are involved in immune defense response. “*” signify genes expressed in the extracellular region

To identify genes that vary with rearing context, we focused on those transcripts that were significantly altered between socialized and non‐socialized flies in multiple species, or overlapped with previously reported gene expression changes in a similar experiment with *D. melanogaster* males^5^ (Figure 5). Approximately, the same number of transcripts were differentially regulated in *D. melanogaster* females as previously reported for *D. melanogaster* males^5^: 201 and 189 genes, respectively, with an approximately 3‐fold greater number of downregulated vs upregulated genes after socialization for both sexes. Of these 201 genes, 81 were differentially regulated in at least 1 other species tested in our assay (Table S3), which is significantly higher than expected by random chance (*p* < 1 × 10^−4^) and supports conservation of experience‐driven transcriptome changes in the brain. Among these conserved changes, immune response genes were significantly enriched (*p* = 5.5*e* ^−4^, GO analysis^44^). The expression levels of this class of genes have previously been observed to vary with multiple types of social experiences in *D. melanogaster,* 5, 35, 37 suggesting a general immune/stress response to social exposure. Genes that encode proteins present in the extracellular region were also enriched (*p* = 6.1*e* ^−10^), which include most of the immune response genes as well as genes involved in the sensory perception of chemical stimuli (Figure 5). One of these genes, *Obp99a*, was significantly downregulated in all 4 species assayed after socialization, which was observed for only 1 other transcript, *CG10621*, a putative selenocystine methyltransferase that has been shown to vary with several different social interactions (Figure 5) and viral infection^61^ in *D. melanogaster*, suggesting it may be part of the immune/stress response. Thirty‐four genes were differentially regulated in at least 3 species, including additional chemosensory genes *a10* and *Obp83b*.

As the behavioral response to socialization varied across species, we were also interested in identifying non‐conserved expression changes that may account for these behavioral differences. Interestingly, the odorant‐binding protein gene *Obp49a* was significantly up‐regulated in *D. erecta* females after socialization, but not in females of any other species. This differential expression in *D. erecta* may contribute to the experience‐mediated change in social space only observed in this species. Social space measurements at higher densities are strongly correlated with *Obp49a* expression levels across species and rearing conditions, further suggesting *Obp49a* is an effector of this phenotype (*R* ^2^ = 0.89 *D. simulans* excluded from regression, Figure 4B). However, when we tested a *D. melanogaster Obp49a* heterozygous mutant in our assay, we did not observe a significant difference in social space. The effect may be specific to *D. erecta* or only realized with more severe alterations in expression, like the observed significant down‐regulation of *Obp49a* in *D. sechellia* relative to the rest of the *melanogaster* subgroup, which exhibits the largest social space at low densities (Figure 1E). *Obp49a* has been shown to mediate the suppression of sugar‐activated gustatory receptor neurons in the presence of bitter chemicals.^38^ As previously mentioned, *D. erecta* responded very differently to food‐containing denatonium than *D. melanogaster*; *D. erecta* clustered in small groups next to bitter food and *D. melanogaster* left immediately after tasting (Figures S3A and S4A). The variation in social space observed with *Obp49a* expression differences in *D. erecta* may mirror changes in aversiveness to chemical stimuli in this species.

## 4 CONCLUSION

While it is clear that accumulated social experience influences behavioral output,^62^ the conservation of this influence has not been systematically studied in a controlled assay. We have perfomed that here by exposing 10 different species of *Drosophilids* to 2 different rearing experiences, and by quantifying the impact of that experience on adult behavior and gene expression levels. Significant shifts in social feeding behaviors and their sensitivity to rearing context, clearly show that these behaviors and their plasticity are highly variable. Determining the mechanism of these interspecific changes is a more difficult task as these differences may be the result of interspecific variations in genic sequences,^14^ anatomy,^45^ pheromone profiles,^52^ and/or neuronal circuitry. The cross‐socialization of *D. melanogaster* and *D. erecta* suggests that at least within the *melanogaster* subgroup, the mechanism of socialization is conserved, but which behaviors are influenced and to what extent is not (Figure 2). The lack of socially mediated plasticity in aggression and social space in the majority of the species does not mean that socialization does not affect any behaviors in these species, just not these behaviors in these contexts. *D. melanogaster* males are much more aggressive than females,^30^ and a significant change in male aggression levels with socialization may be more conserved across these species.

Clearly, there is some conserved response to socialization within the *melanogaster* subgroup, as there is significant overlap in differentially expressed genes after prolonged social exposure (Figure 5). A growing body of evidence points to evolved differences in the sequence and expression of chemosensory genes as a driving force in behavioral evolution.^55^, ^56^, ^57^ This is likely because variations in odorant/pheromone detection at the most peripheral level of the nervous system can immediately alter the degree of influence of a given stimuli, or which neurons are responsive to that stimuli. Our observation that these genes are enriched among those differentially regulated in *D. erecta* and *D. sechellia*, the 2 species that display extremes of all behaviors assayed, supports this idea. Observed differences in the regulation of these genes in response to social experience, likely contributes to the observed differences in behavioral plasticity. Further experiments are needed to directly link expression changes observed here to behavioral shift, but our data will be instrumental in guiding those efforts.

## Supporting information


**Video S1** *Drosophila erecta* interacting on food. Video of *D. erecta* interacting on food source. Flies with colored paint were raised in isolation and have not seen another fly prior to being loaded into the aggregation chamber. The white‐painted fly was raised in a group of 25 flies to control for the effect of paint on behavior

**Video S2** *Drosophila melanogaster* interacting on food. Video of *D. melanogaster* interacting on food source. Flies with colored paint were raised in isolation and have not seen another fly prior to being loaded into the aggregation chamber. Note hyperaggressiveness of the orange painted fly

**TABLE S1** Ecological and strain information for 10 species

**TABLE S2** Differentially expressed genes among melanogaster subgroup. Genes that are significantly differentially expressed in 1 species in the *melanogaster* subgroup vs all other species in the *melanogaster* subgroup. Data for each species are given on separate pages within the file. “id”—*Drosophila melanogaster* ortholog gene name. “Mean”—average expression value for that gene as determined by DESeq.^42^ “log2FC”—the log2‐fold change of the reference species relative to the species designated for that column. “FB name”—FlyBase reference name.^41^ “Full name”—Full name of *D. melanogaster* ortholog. “Bio process”—annotated biological process for that gene from FlyBase^41^

**TABLE S3** Differentially expressed genes between socialized and non‐socialized flies. “id”—*Drosophila melanogaster* ortholog gene name. “MeanS”—Mean expression in socialized flies as determined by DESeq.^42^ “MeanN”—Mean expression in Non‐socialized flies. “log2FC”—the log2‐fold change of MeanN/MeanS. “padj”—adjusted *P*‐value showing significance of differential expression between socialized and non‐socialized flies as determined by DESeq.^42^ “FB name”—FlyBase reference name.^41^ “Full name”—Full name of *D. melanogaster* ortholog. “Species”—which other species this gene was significantly differentially regulated between Socialized and Non‐socialized rearing conditions: “m” is *D. melanogaster*, “e” is *Drosophila erecta*, “s” is *Drosophila simulans* and “y” is *Drosophila yakuba*. “Bio process” —annotated biological process for that gene from FlyBase^41^

**TABLE S4** RNA‐Seq summary statistics. “Sample”—name of biological replicate. The 3 letters are the first 3 letters of the species name. If the name ends in “N”, that is data for that species reared in isolation. “*R* ^2^” values are the correlation between that replicate and all biological replicates below it. Correlations were perfomed over log‐normalized data: log(RPKM + 1)

**TABLE S5** All reads for *Drosophila melanogaster* orthologs in all species. Raw reads, DESeq Normalized reads,^42^ RPKM values and TPM values for all *D. melanogaster* orthologs for all biological replicates

**TABLE S6** Raw data for all figures. Raw data used to generate all figures and supplemental figures

**TABLE S7** R script to identify and count files. Core R functions to identify and count files on the food source over time

**FIGURE S1** Flies show slight but non‐significant preference for native food type. Representative images taken at 120 minutes for 3 species of flies (rows) assayed with 3 types of food (columns). Bar graphs show the average number of flies on the food at 120 minutes ±1 SEM from 4 replicates for each condition

**FIGURE S2** T‐40 varies as a function of population size in a cooperative model. The time it takes 40% of the flies in a population to arrive at a food source (T40) in a cooperative (red) and non‐cooperative (blue: free diffusion) food search simulation. We report the mean and SD of 100 simulations for each condition

**FIGURE S3** *Drosophila melanogaster* exhibit cooperativity in food choice assay. (A) Representative snapshots of flies accumulated on food sources with and without denatonium. (B) Line graphs plot the number of new flies that went to the food containing denatonium (red) and the food without (blue) over a 21 minute interval. Each plot is a different experimental replicate. (C) Bar graphs show the cumulative number of flies for each replicate that went to each food source over 84 minutes

**Figure S4** *Drosophila erecta* cluster next to food containing denatonium. (A) Snapshots of *D. erecta* aggregated on food sources without and with denatonium (top and bottom pictures respectively) for 4 experimental replicates. (B) The average number of flies accumulated on food with denatonium (red) and without (blue) ±1 SEM as a function of time

**FIGURE S5** Food odor is sufficient to drive *Drosophila erecta* aggregation, but not *Drosophila melanogaster*. (A) Snapshots of fly aggregation on food or food and 50 flies covered by either a cheesecloth (left 4 images) or coverslip (right 4 images). (B) The average number of flies that aggregated for each testing condition ±1 SD after 2 hours.

**FIGURE S6** Social space varies with fly density and between species. The mean Social Space (*y*‐axis) was calculated between all flies in the frame, and plotted against the number of flies present (*x*‐axis). Social Space values were averaged over bin sizes of 10 flies (5‐15 flies, 15‐25, 25‐25, etc.), and plotted at the lower density for each bin; data at point 5 is for densities of 5 to 15 flies. Error bars are SE of the mean. The average body length for each species is given in pixels next to that species' name in the legend

**FIGURE S7** Species food location preference. Images on the left are snapshots of flies aggregated on the food after 120 minutes. Graphs on the right show what fraction of the flies in the frame are on the food (*y*‐axis) or next to the food (*x*‐axis). Each point represents a single processed frame. The red line is *y* = *x*, and points on the red line signify all flies in that frame were on the food source.

**FIGURE S8** Instance of aggression does not correlate with number of flies at food. The number of aggressive lunges exhibited by individual flies within first 150 seconds of arriving to the food (*y*‐axis) was plotted against the number of flies in the frame when that fly arrived (*x*‐axis) for each species. Data for each species were colored according to legend. Regression lines (dashed lines) were fit to the data for each species, and the *R* ^2^ value is reported in the legend next to the species name

**Figure S9** *Z*‐score distributions for individual, painted flies. Each graph represents a different experimental replicate for that species. The color of each curve is the color of paint on that fly. Black curves are data for white‐painted, socially reared flies. *Z*‐score distributions are calculated as described in Section 2
