# Supplementary material for: Conservation of the behavioral and transcriptional response to social experience among Drosophilids
Source: Genes Brain Behav. 2018 Jul 9;18(1):e12487. doi: 10.1111/gbb.12487 (PMC7379240; doi:10.1111/gbb.12487)
Supplement: Supplementary file 10 — FIGURE S1 Flies show slight but non‐significant preference for native food type. Representative images taken at 120 minutes for 3 species of flies (rows) assayed with 3 types of food (columns). Bar graphs show the average number of flies on the food at 120 minutes ±1 SEM from 4 replicates for each condition [file GBB-18-e12487-s005.pdf]

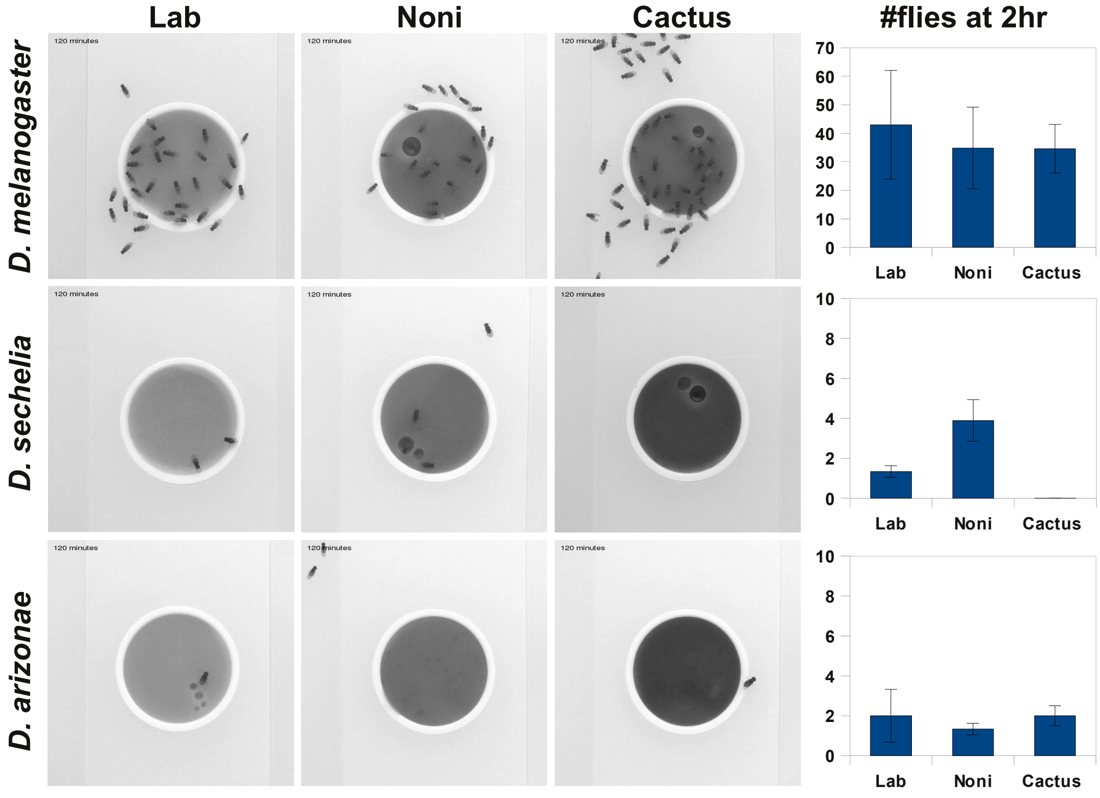

Supplemental Figure 1: Flies show slight but non-significant preference for native food type. Representative images taken at 120 minutes for three species of flies (rows) assayed with three types of food (columns). Bar graphs show the average number of flies on the food at 120 minutes $\pm$ 1 SEM from 4 replicates for each condition.
