# Supplementary material for: Conservation of the behavioral and transcriptional response to social experience among Drosophilids
Source: Genes Brain Behav. 2018 Jul 9;18(1):e12487. doi: 10.1111/gbb.12487 (PMC7379240; doi:10.1111/gbb.12487)
Supplement: Supplementary file 11 — FIGURE S2 T‐40 varies as a function of population size in a cooperative model. The time it takes 40% of the flies in a population to arrive at a food source (T40) in a cooperative (red) and non‐cooperative (blue: free diffusion) food search simulation. We report the mean and SD of 100 simulations for each condition [file GBB-18-e12487-s010.pdf]

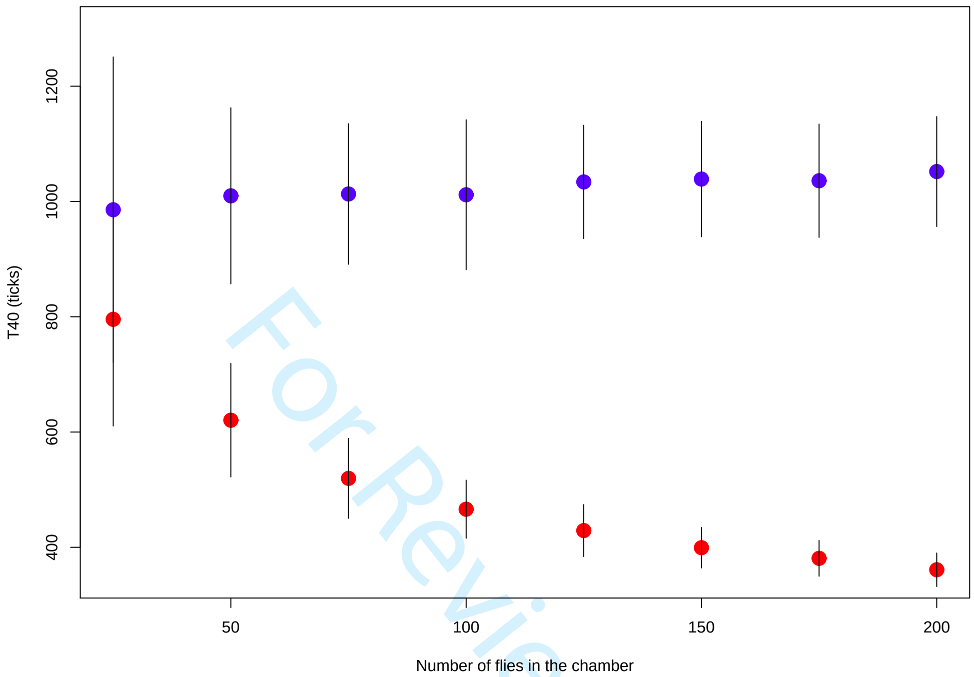

For Review Only

Supplemental Figure 2: T-40 varies as a function of population size in a cooperative model. The time it takes 40% of the flies in a population to arrive at a food source (T40) in a cooperative (red) and non-cooperative (blue: free diffusion) food search simulation. We report the mean and standard deviation of 100 simulations for each condition.
