# Supplementary material for: Conservation of the behavioral and transcriptional response to social experience among Drosophilids
Source: Genes Brain Behav. 2018 Jul 9;18(1):e12487. doi: 10.1111/gbb.12487 (PMC7379240; doi:10.1111/gbb.12487)
Supplement: Supplementary file 12 — FIGURE S3 Drosophila melanogaster exhibit cooperativity in food choice assay. (A) Representative snapshots of flies accumulated on food sources with and without denatonium. (B) Line graphs plot the number of new flies that went to the food containing denatonium (red) and the food without (blue) over a 21 minute interval. Each plot is a different experimental replicate. (C) Bar graphs show the cumulative number of flies for each replicate that went to each food source over 84 minutes [file GBB-18-e12487-s013.pdf]

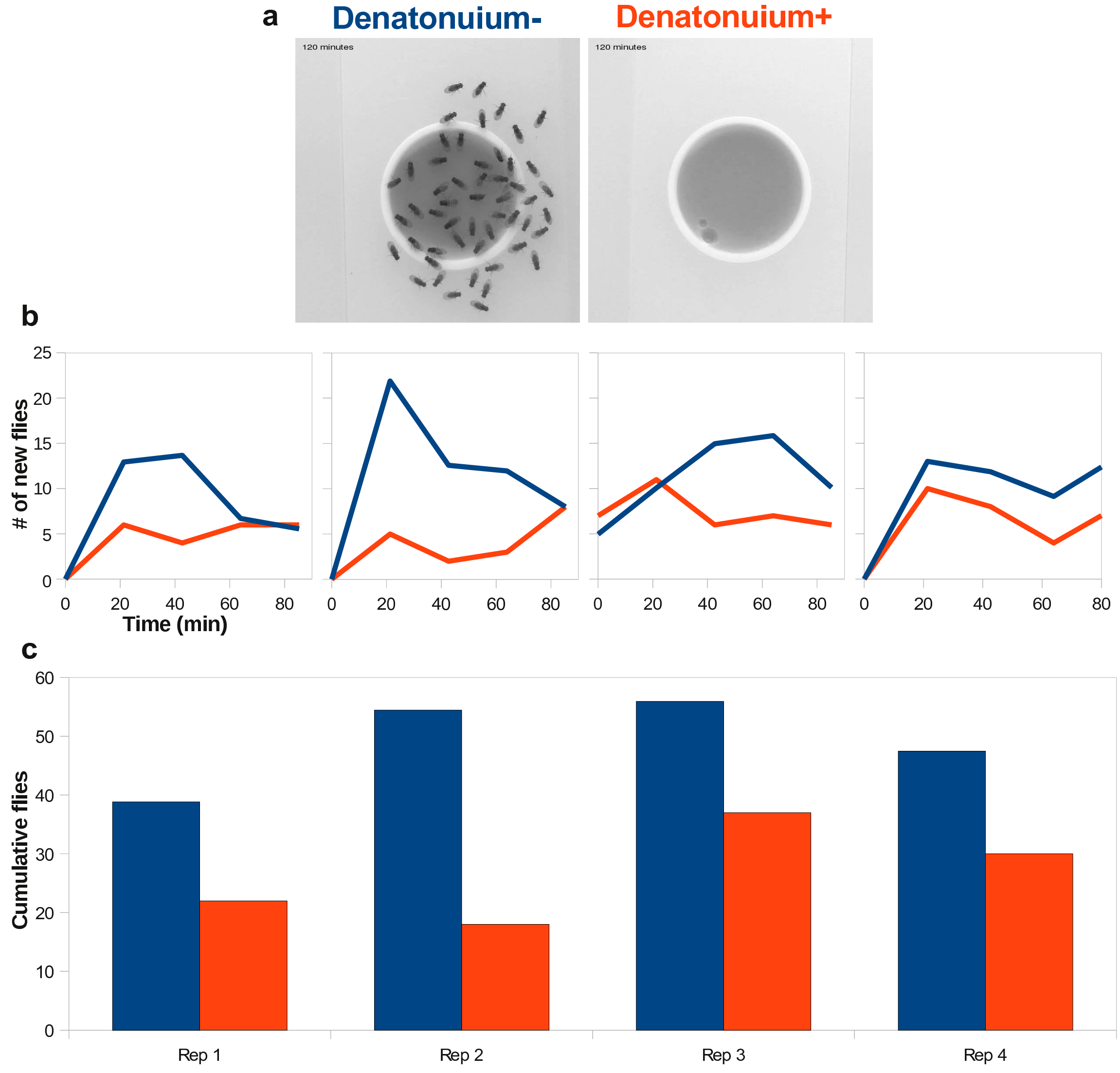

Supplemental Figure 3: *D. melanogaster* exhibit cooperativity in food choice assay. (a) Representative snapshots of flies accumulated on food sources with and without denatonium. (b) Line graphs plot the number of new flies that went to the food containing denatonium (Red) and the food without (Blue) over a 21 minute interval. Each plot is a different experimental replicate. (c) Bar graphs show the cumulative number of flies for each replicate that went to each food source over 84 minutes.
