# Supplementary material for: Conservation of the behavioral and transcriptional response to social experience among Drosophilids
Source: Genes Brain Behav. 2018 Jul 9;18(1):e12487. doi: 10.1111/gbb.12487 (PMC7379240; doi:10.1111/gbb.12487)
Supplement: Supplementary file 13 — Figure S4 Drosophila erecta cluster next to food containing denatonium. (A) Snapshots of D. erecta aggregated on food sources without and with denatonium (top and bottom pictures respectively) for 4 experimental replicates. (B) The average number of flies accumulated on food with denatonium (red) and without (blue) ±1 SEM as a function of time [file GBB-18-e12487-s011.pdf]

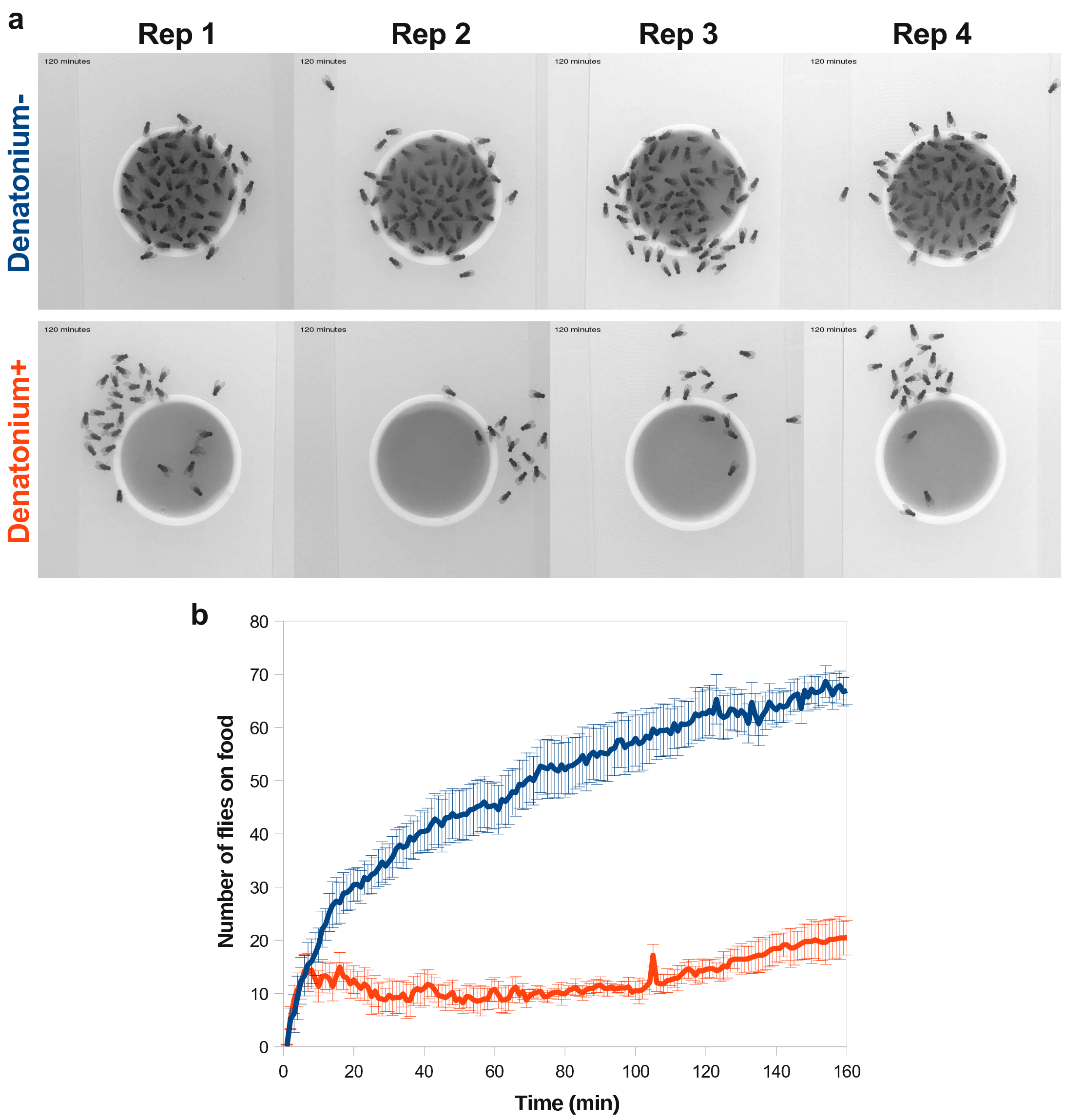

Supplemental Figure 4: *D. erecta* cluster next to food containing denatonium. (a) Snapshots of *D. erecta* aggregated on food sources without and with denatonium (top and bottom pictures respectively) for 4 experimental replicates. (b) The average number of flies accumulated on food with denatonium (Red) and without (Blue) ± 1 SEM as a function of time.
