# Supplementary material for: Conservation of the behavioral and transcriptional response to social experience among Drosophilids
Source: Genes Brain Behav. 2018 Jul 9;18(1):e12487. doi: 10.1111/gbb.12487 (PMC7379240; doi:10.1111/gbb.12487)
Supplement: Supplementary file 14 — FIGURE S5 Food odor is sufficient to drive Drosophila erecta aggregation, but not Drosophila melanogaster. (A) Snapshots of fly aggregation on food or food and 50 flies covered by either a cheesecloth (left 4 images) or coverslip (right 4 images). (B) The average number of flies that aggregated for each testing condition ±1 SD after 2 hours. [file GBB-18-e12487-s008.pdf]

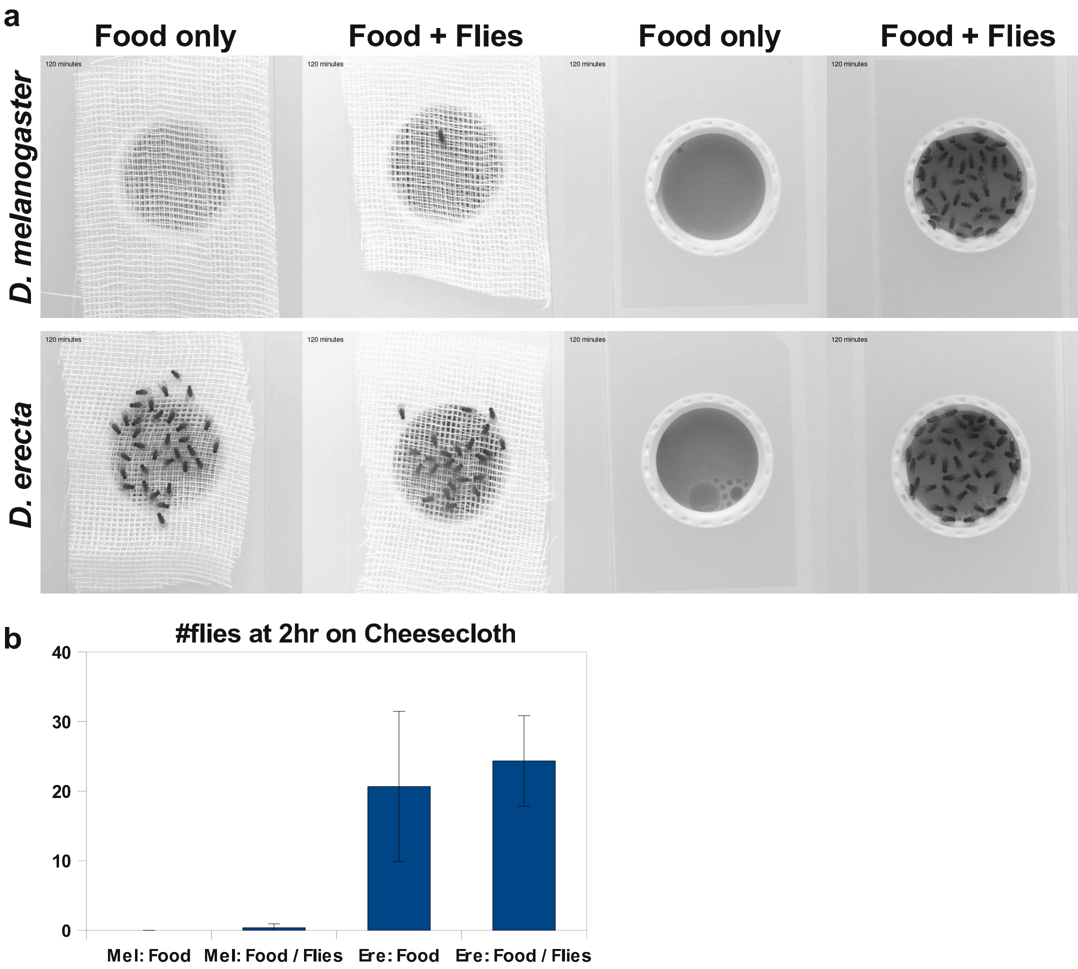

Supplemental Figure 5: Food odor is sufficient to drive *D. erecta* aggregation, but not *D. melanogaster*.

(a) Snapshots of fly aggregation on food or food and 50 flies covered by either a cheesecloth (left 4 images) or coverslip (right 4 images). (b) The average number of flies that aggregated for each testing condition $\pm$ 1 SD after 2 hours.
