# Supplementary material for: Conservation of the behavioral and transcriptional response to social experience among Drosophilids
Source: Genes Brain Behav. 2018 Jul 9;18(1):e12487. doi: 10.1111/gbb.12487 (PMC7379240; doi:10.1111/gbb.12487)
Supplement: Supplementary file 15 — FIGURE S6 Social space varies with fly density and between species. The mean Social Space (y‐axis) was calculated between all flies in the frame, and plotted against the number of flies present (x‐axis). Social Space values were averaged over bin sizes of 10 flies (5‐15 flies, 15‐25, 25‐25, etc.), and plotted at the lower density for each bin; data at point 5 is for densities of 5 to 15 flies. Error bars are SE of the mean. The average body length for each species is given in pixels next to that species' name in the legend [file GBB-18-e12487-s017.pdf]

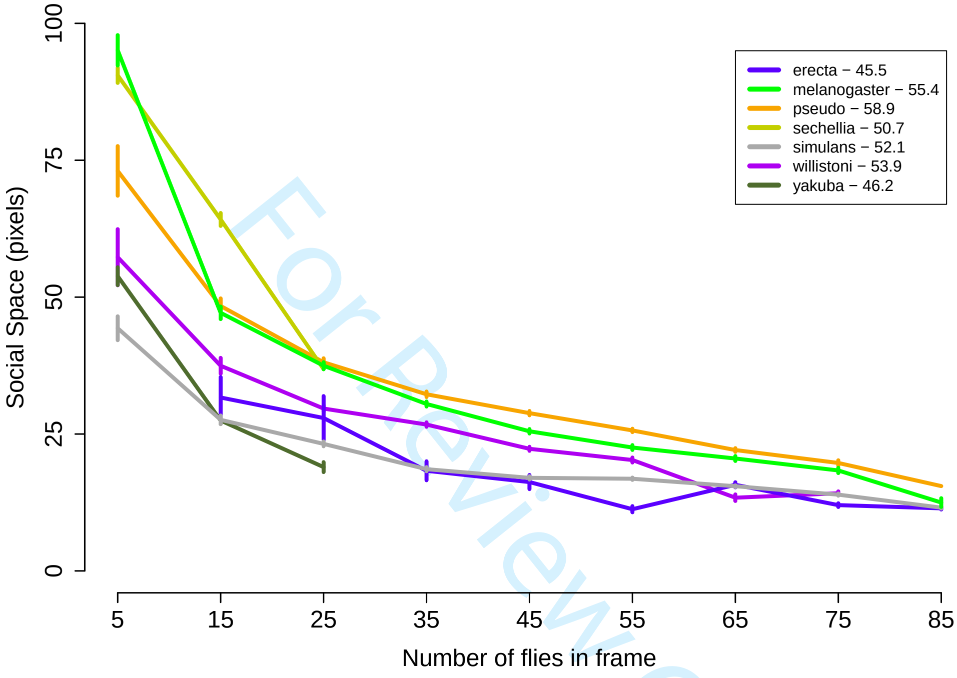

For Review Only

Supplemental Figure 6: Social space varies with fly density and between species. The mean Social Space (y-axis) was calculated between all flies in the frame, and plotted against the number of flies present (x-axis). Social Space values were averaged over bin sizes of 10 flies (5-15 flies, 15-25, 25-25, etc.), and plotted at the lower density for each bin; data at point 5 is for densities of 5-15 flies. Error bars are standard error of the mean. The average body length for each species is given in pixels next to that species' name in the legend.
