# Supplementary material for: Conservation of the behavioral and transcriptional response to social experience among Drosophilids
Source: Genes Brain Behav. 2018 Jul 9;18(1):e12487. doi: 10.1111/gbb.12487 (PMC7379240; doi:10.1111/gbb.12487)
Supplement: Supplementary file 16 — FIGURE S7 Species food location preference. Images on the left are snapshots of flies aggregated on the food after 120 minutes. Graphs on the right show what fraction of the flies in the frame are on the food (y‐axis) or next to the food (x‐axis). Each point represents a single processed frame. The red line is y = x, and points on the red line signify all flies in that frame were on the food source. [file GBB-18-e12487-s003.pdf]

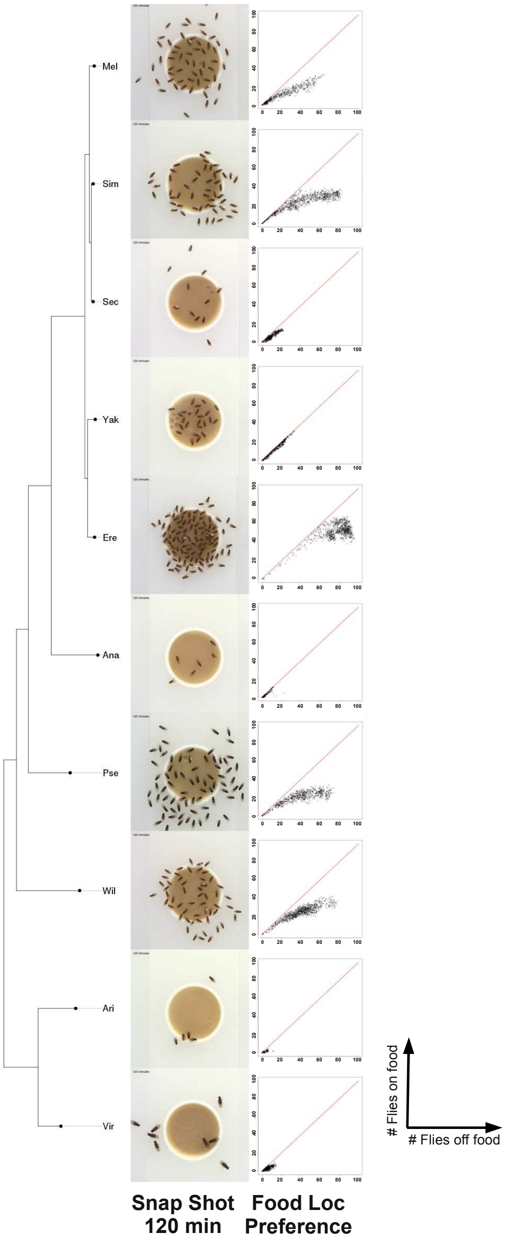

Supplemental Figure 7: Species food location preference.

Images on the left are snapshots of flies aggregated on the food after 120 minutes. Graphs on the right show what fraction of the flies in the frame are on the food (y-axis) or next to the food (x-axis). Each point represents a single processed frame. The red line is y=x, and points on the red line signify all flies in that frame were on the food source.
