# Supplementary material for: Conservation of the behavioral and transcriptional response to social experience among Drosophilids
Source: Genes Brain Behav. 2018 Jul 9;18(1):e12487. doi: 10.1111/gbb.12487 (PMC7379240; doi:10.1111/gbb.12487)
Supplement: Supplementary file 17 — FIGURE S8 Instance of aggression does not correlate with number of flies at food. The number of aggressive lunges exhibited by individual flies within first 150 seconds of arriving to the food (y‐axis) was plotted against the number of flies in the frame when that fly arrived (x‐axis) for each species. Data for each species were colored according to legend. Regression lines (dashed lines) were fit to the data for each species, and the R 2 value is reported in the legend next to the species name [file GBB-18-e12487-s018.pdf]

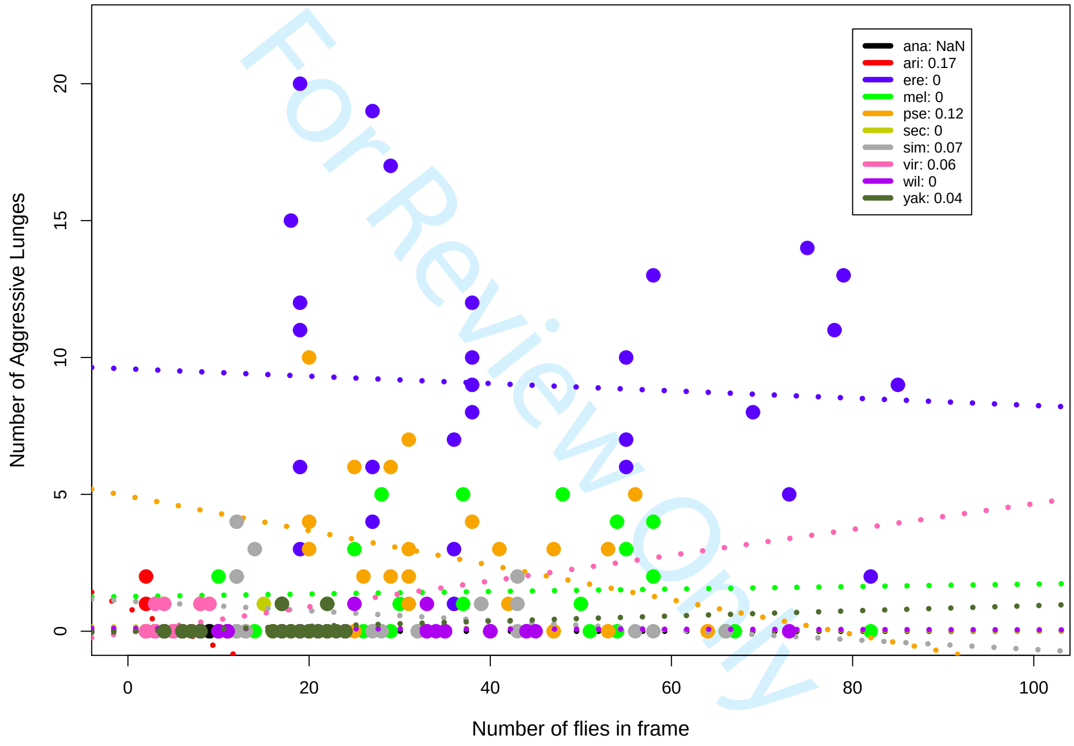

Supplemental Figure 8: Instance of aggression does not correlate with number of flies at food.

The number of aggressive lunges exhibited by individual flies within first 150 seconds of arriving to the food (y-axis) was plotted against the number of flies in the frame when that fly arrived (x-axis) for each species. Data for each species were colored according to legend. Regression lines (dashed lines) were fit to the data for each species, and the R^2 value is reported in the legend next to the species name.
