# Supplementary material for: Conservation of the behavioral and transcriptional response to social experience among Drosophilids
Source: Genes Brain Behav. 2018 Jul 9;18(1):e12487. doi: 10.1111/gbb.12487 (PMC7379240; doi:10.1111/gbb.12487)
Supplement: Supplementary file 18 — Figure S9 Z‐score distributions for individual, painted flies. Each graph represents a different experimental replicate for that species. The color of each curve is the color of paint on that fly. Black curves are data for white‐painted, socially reared flies. Z‐score distributions are calculated as described in Section 2 [file GBB-18-e12487-s004.pdf]

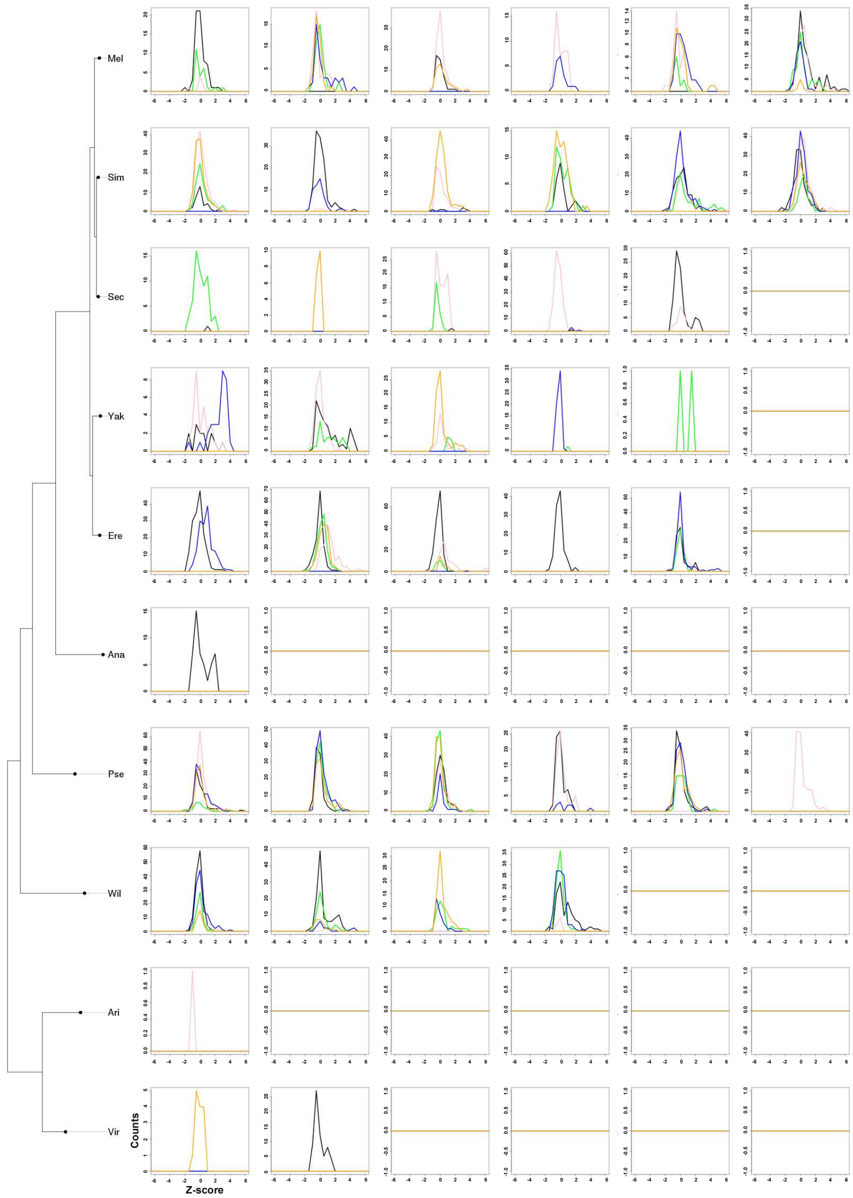

Supplemental Figure 9: Z-score distributions for individual, painted flies. Each graph represents a different experimental replicate for that species. The color of each curve is the color of paint on that fly. Black curves are data for white-painted, socially reared flies. Z-score distributions are calculated as described in Materials and Methods.
